# Sweat and heat production related to air humidity during exercise and inactive recovery - a laboratory study

**DOI:** 10.1186/2046-7648-4-S1-A146

**Published:** 2015-09-14

**Authors:** Erik U Høye, Mariann Sandsund, Randi Eidsmo Reinertsen

**Affiliations:** 1 SINTEF Technology and Society, Department of Health Research, Trondheim, Norway; 2 Norwegian University of Science and Technology, Department of Biology, Trondheim, Norway

## Introduction

Certain occupational groups are exposed to unfavourable work conditions, such as exposure to warm and humid environments and alternations between high and low work intensity. Such conditions affect thermoregulatory responses as well as thermal sensation and comfort. Exercise and work capacity at moderate intensity in a warm environment are progressively impaired as relative humidity (rh) increases [1]. However, the influence of relative humidity on regional sweat rate (RSR) during high-intensity work followed by a recovery period has not been studied. This study examines the relationship between rh and RSR during inactive recovery after a period of high work intensity.

## Methods

We measured RSR in 10 healthy male subjects aged between 20 and 30 by means of absorbent pads, in two trials performed at SINTEF's Work Physiology Laboratory. The trials consisted of running for 20 min at 68(4) % VO_2max _followed by 30 min of inactive recovery at 19 and 85 % rh at 30 °C and 0(0.2) m.s^-1 ^air velocity. Nude body mass, rectal (T_re_) and local skin (T_s_) (6 sites) temperatures (YSI 400 (0.15) °C), heart rate (HR) (Polar S810™ Electro OY), oxygen consumption (Oxycon Pro, Cardinal Health) and RSR on the central mid-back and posterior-forearm (Air Laid, Meditas) were measured. The method of measuring RSR by means of absorbent pads was modified from Smith and Havenith [2]. Starting with the last five minutes of running, the absorbent pads were changed every five min. After the inactive recovery phase, nude body mass was measured again in order to estimate gross sweat loss (GSL).

## Results

GSL was higher at 85 % rh (796(414) g.h^-1^) than at 19 % rh (489(140) g.h^-1^) (p < 0.05). RSR on the back was 1105(95 % CI, 691 to 1765) g.m^-2^.h^-1 ^during the first five min of recovery at 85 % rh. At 19 % rh, RSR was 675(417 to 1093) g.m^-2^.h^-1 ^for the same time interval, which was significantly lower (p < 0.05). RSR on the back fell to 395(227 to 686) and 165(113 to 240) g.m^-2^.h^-1 ^at 85 % and 19 % rh during the last five min of recovery (p < 0.05). The corresponding RSR on the arm was 459(573 to 367) and 225(171 to 296) g.m^-2^.h^-1 ^at 85 % rh, and 216(155 to 300) and 16(6 to 48) g.m^-2^.h^-1 ^at 19 % rh (p < 0.05). RSR showed weak to strong correlations with T_s _during recovery, but not during exercise. T_re _continued to increase for seven and three minutes post-exercise at 85 % and 19 % rh. HR was 11 b.min^-1 ^higher after exercise and during the first twenty min of recovery at 85 % compared to 19 % rh (p < 0.05).

## Discussion

The differences in RSR were in accordance with the results of Smith and Havenith [2]. Our findings indicate a prolonged sweat response at 85 % rh compared to 19 % rh. This corresponds to the continued increase in T_re_, T_s _and a higher HR post-exercise at 85 % rh, indicating a higher thermal load.

## Conclusion

Thermal load measured by RSR, HR, T_re _and T_s _was higher during post-exercise inactive recovery at 85 % rh compared to 19 % rh at 30 °C. This study emphasizes the importance of including the effect of rh in assessments of work in hot environments.
